# Sexual Selection and the Evolution of Male Reproductive Traits in Benthic Octopuses

**DOI:** 10.3389/fphys.2019.01238

**Published:** 2019-10-09

**Authors:** Christian M. Ibáñez, Javiera Pérez-Álvarez, Jennifer Catalán, Sergio A. Carrasco, M. Cecilia Pardo-Gandarillas, Enrico L. Rezende

**Affiliations:** ^1^Departamento de Ecología y Biodiversidad, Facultad de Ciencias de la Vida, Universidad Andres Bello, Santiago, Chile; ^2^Millennium Nucleus for Ecology and Sustainable Management of Oceanic Islands (ESMOI), Coquimbo, Chile; ^3^Departamento de Biología Marina, Facultad de Ciencias del Mar, Universidad Católica del Norte, Coquimbo, Chile; ^4^Departamento de Ciencias Ecológicas, Facultad de Ciencias, Universidad de Chile, Santiago, Chile; ^5^Departamento de Ecología, Facultad de Ciencias Biológicas, Pontificia Universidad Católica de Chile, Santiago, Chile; ^6^Center of Applied Ecology and Sustainability (CAPES), Santiago, Chile

**Keywords:** Octopodoidea, sexual selection, sperm competition, cryptic choice, spermatophores, hectocotylus, ligula, phylogeny

## Abstract

Competition between same-sex organisms, or intra-sexual selection, can occur before and after mating, and include processes such as sperm competition and cryptic female choice. One of the consequences of intra-sexual selection is that male reproductive traits tend to evolve and diverge at high rates. In benthic octopuses, females often mate with more than one male in a single reproductive event, opening the venue for intra-sexual selection at multiple levels. For instance, males transfer spermatophores through hectocotylus, and can remove the spermatophores left by other males. Considering the limited evidence on post-copula competition in benthic octopuses, and the potential to affect the evolution of reproductive traits within octopodids, we put this hypothesis to a test employing a phylogenetic comparative approach. We combined data on hectocotylized arm length (HAL), ligula length (LL), spermatophore length (SL) with a Bayesian molecular phylogeny of 87 species, to analyze how reproductive traits have diverged across lineages and covary with body size (mantle length; ML). First, additionally to ML, we estimated the phylogenetic signal (λ) and mode of evolution (κ) in each reproductive trait. Second, we performed phylogenetic regressions to quantify the association among reproductive traits and their co-variation with ML. This analysis allowed us to estimate the phenotypic change along a branch into the phylogeny, and whether selection may have played a role in the evolution and diversification of specific clades. Estimations of λ were always high (>0.75), indicating concordance between the traits and the topology of the phylogenetic tree. Low values of κ (<1.0) suggested that evolution depends on branch lengths. All reproductive traits exhibiting a positive relation with ML (β > 0.5 in all cases). Overall, evolutionary rate models applied to the SL-ML regression suggested that octopuses of the family Megaleledonidae have evolved larger spermatophores than expected for their size. The regression HAL-ML indicated that HAL was more variable in Megaleledonidae than in the remaining clades, suggesting that the high divergence across species within this group might partially reflect intra-sexual selection. These results support the hypothesis that, at least in some lineages, sexual selection may account for the divergence in reproductive traits of male octopuses.

## Introduction

The evolution of mate choice and mating competition has been a major component of Darwin’s theory of sexual selection (Darwin, 1871). Since then, it has been generally accepted that different selective pressures acting on male and female attributes may give rise to sexually dimorphic traits, which are often interpreted as evidence of direct competition for mates within a given sex, differential success to attract potential mates from the opposite sex, or gametic competition (see Basolo, 1990; Birkhead, 1992; Evans and Sherman, 2013). At the gametic level, competition can occur before and after mating through sperm competition or cryptic female choice. Whereas sperm competition involves strategies by the male to either remove, displace or inhibit the sperm of other males, cryptic female choice constitutes female-biased selection through the use/removal of sperm to fertilize their eggs. Competition at the gametic level has been described in organisms from several phyla, such as insects, molluscs, birds and mammals (Mann, 1984), and is generally enhanced in polyandric species where females can mate with multiple males in a single reproductive episode (Birkhead, 1998; Gomendio, 2002). Because of its relevance to males’ reproductive success (Eberhard, 1998; Snook, 2005), multiple responses have evolved to outcompete rival males, including: (i) the production of spermatophores or packages of sperm (Mann, 1984; Nigmatullin et al., 2003) that, when transferred to females, may occupy considerable space within the storage organs preventing other spermatophores from being stored (Thornhill and Alcock, 1983), (ii) the removal of other males’ spermatophores during copulation (Cigliano, 1995), or (iii) the production of sperm with inhibitory effects on the rival males’ sperm function (Snook, 2005). Because of this evolutionary arms race, the genitalia of several organisms exhibit extreme differences in size and shape across closely related species and are presumed to evolve faster than other traits (Eberhard, 1985; Genevcius et al., 2017).

**FIGURE 1:**
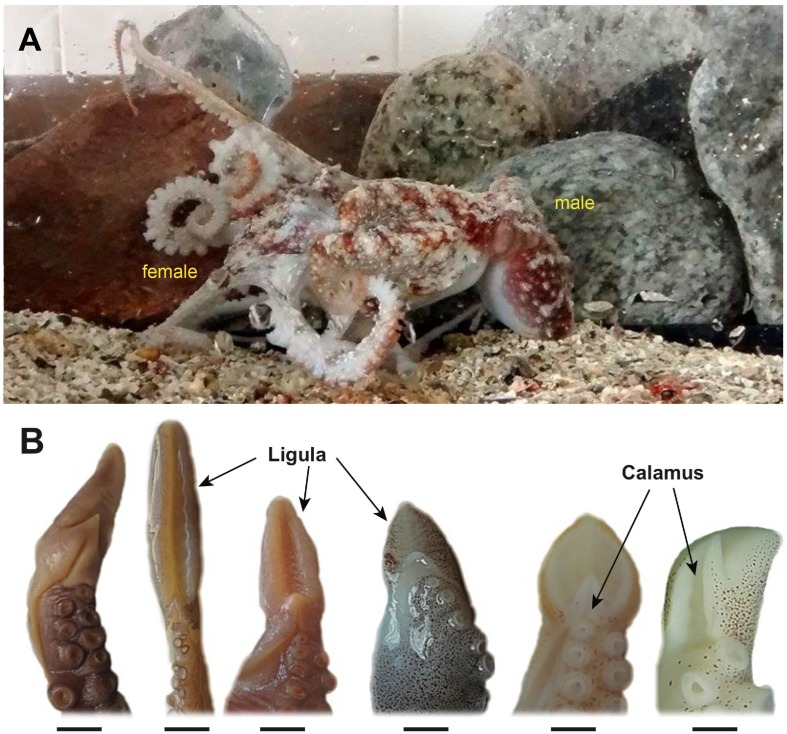
Mating behavior and reproductive organs in benthic octopuses. **(A)** Mounting during the copula stage, in the small-sized benthic octopus *Robsonella fontaniana*, where male transfers spermatophores into the females’ mantle cavity using the modified arm called hectocotylus. **(B)** Morphological diversity of benthic octopus hectocotyli, showing the differentiated ligula and calamus. From left to right: *Muusoctopus tangaroa*, *Pinnoctopus cordiformis*, *Muusoctopus longibrachus*, *Graneledone taniwha*, *Octopus huttoni*, *Octopus mernoo* (Photo credits: SC and CI, respectively).

Polyandry, sexual dimorphism and sexual selection have been described in several lineages of cephalopod molluscs and is widespread in this group (Mann, 1984; Hanlon and Messenger, 1996; Squires et al., 2012). Polyandrous behavior has been observed in female octopuses, potentially increasing post-mating sexual selection and driving the evolution of a myriad of sperm transfer strategies (e.g., male mounting female; Figure 1A; for other examples see Hanlon and Messenger, 1996; Cheng and Caldwell, 2000; Huffard et al., 2008; Gutierrez et al., 2012; Morse and Huffard, 2019). In all cases, male octopuses pack their sperm into spermatophores and transfer them to females by using a modified arm called hectocotylus. This specialized arm employed to deposit the sperm packages into the females’ pallial cavity is characterized by two well defined segments, the calamus and the ligula (see Hanlon and Messenger, 1996; Wodinsky, 2008; Marian, 2015), and by a considerable inter-specific morphological variation (Figure 1B). This variation has been associated with the successful transference of spermatophores during mating (Robson, 1926); however, direct behavioral evidence on their role in removing or breaking down spermatophores from rival males remain speculative (Cigliano, 1995; Hanlon and Messenger, 1996; Norman et al., 2004), providing an important framework for evaluating untested hypotheses on sexual selection (see Voight, 2009). Furthermore, spermatophores also exhibit considerable inter-specific differences in size (e.g., ranging from 7 to 1130 mm in length), even after accounting for size effects (Voight, 2009). This is likely because individuals with larger spermatophores have greater sperm reservoirs and consequently much more sperm to fertilize females’ eggs (Voight, 2001). Nonetheless, in spite of the high levels of morphological variation in these traits, the evolution of hectocotyli and spermatophores across benthic octopuses remains poorly understood, as previous comparative studies have not accounted for the evolutionary history of the lineages involved (see Voight, 2001, 2002, 2009).

Here, we study the evolution and diversification of these reproductive traits across benthic octopuses, employing phylogenetic analytical methods that take into consideration patterns of relatedness between different lineages. Phylogenetic methods are currently indispensable to understand patterns of phenotypic diversification and their underlying processes, as well as the direction and magnitude of inferred evolutionary changes (Felsenstein, 1985; Harvey and Pagel, 1991; Rezende and Diniz-Filho, 2012). Accordingly, recent phylogenetic comparative studies have been quite successful in reconstructing the evolution of life-history strategies in cephalopods in response to different environmental pressures (see Lindgren et al., 2012; Ibáñez et al., 2014, 2018; Pardo-Gandarillas et al., 2018). In the present work, we used a phylogenetic approach to: (a) reconstruct how hectocotyli and spermatophores have evolved along a molecular phylogeny including 87 species of benthic octopuses, (b) explore the correlated evolution between these traits and body size, and (c) employ variable-rates phylogenetic regression to determine which clades exhibit abnormally high rates of phenotypic evolution in response to selection. Even though results could be interpreted as putative evidence of strong post-copulatory sexual selection, we also discuss alternative adaptive scenarios that might have given rise to the observed differences across lineages.

## Materials and Methods

### Dataset and Phylogeny

**FIGURE 2:**
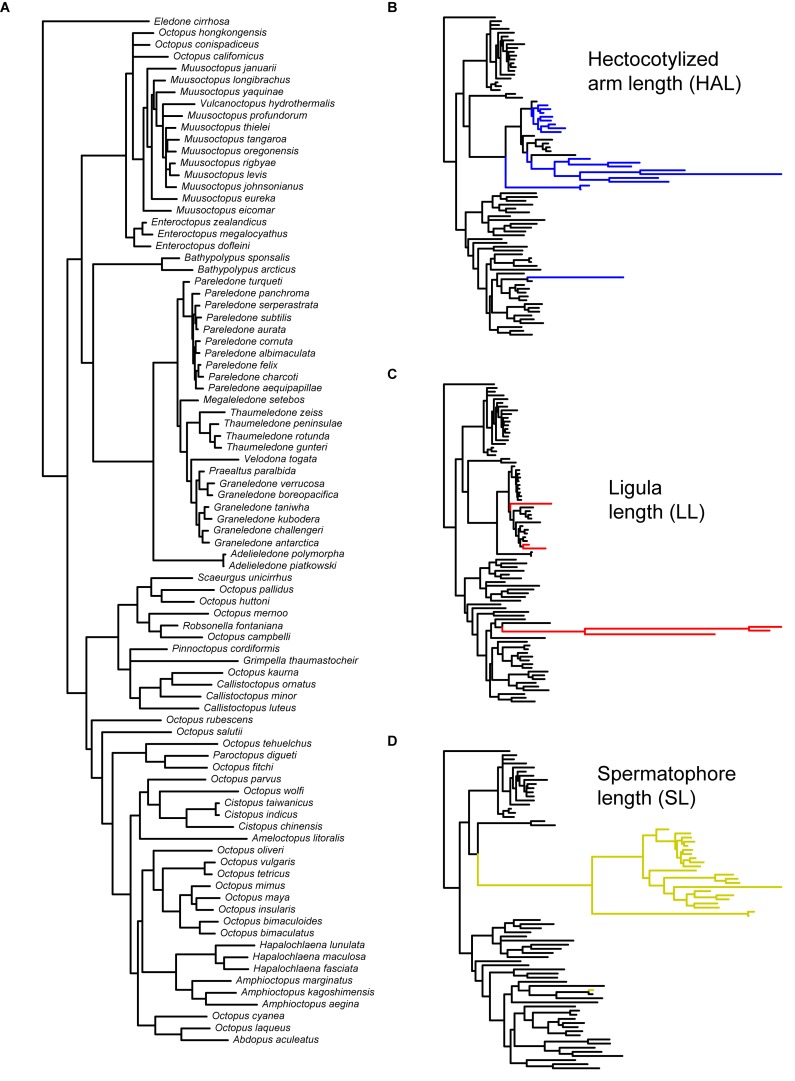
Phylogeny of benthic octopuses and reconstructed phenotypic evolution of reproductive traits. **(A)** Phylogenetic hypothesis employed in this study (*n* = 87 spp.), with branch length proportional to the DNA sequence divergence (see section “Materials and Methods”). Results of the variable-rates regression model for **(B)** hectocotylized arm length (*n* = 84 spp.), **(C)** ligula length (*n* = 86 spp.), and **(D)** spermatophore length (*n* = 78 spp.). Branch lengths in these panels are scaled to estimated phenotypic change, and colored branches indicate regions in which positive selection is detected (Δ_V_/Δ_B_ > 2.0).

We performed an extensive literature review to obtain information on the variability of reproductive traits across different lineages of benthic octopuses. For our analyses, we selected descriptive measures that have been extensively studied with relatively standardized protocols and that could, therefore, be readily compared across different studies (see Table 1): mantle length (ML), arm length (AL), ligula length (LL), hectocotylized arm length (HAL), and spermatophores length (SL). Subsequently, we combined this information with a new phylogenetic hypothesis of benthic octopuses encompassing a total of 97 species, including outgroups (Mendeley Datasets: doi: 10.17632/5vkm46hm49.1), that are based on three mitochondrial genes (16S ribosomal RNA, Cytochrome oxidase I, Cytochrome oxidase III) and one nuclear gene (Rhodopsin). A detailed explanation regarding the analyses underlying the phylogenetic reconstruction, estimation of uncertainty and validation of our working phylogeny has been provided elsewhere (Ibáñez et al., 2018). Briefly, phylogenetic relationships were inferred from a partitioned matrix (16S, COI + COIII, RHO) with a different substitution model for each gene. This matrix was composed of 97 species, including 88 species from the superfamily Octopodoidea, and two species from the superfamily Argonautoidea, six cirrates and the vampire squid *Vampyroteuthis infernalis* as outgroups. Bayesian analyses were conducted using MrBayes 3.2 with four chains, each with ten million generations, sampled every 1,000 generations. The first 1,000 trees of each run were discarded as burn-in, and a consensus of the remaining trees was calculated. For simplicity, we have excluded the outgroups, providing the reconstructed relationships for the 87 benthic species in the dataset (excluding *Vitreledonella richardi*, Figure 2A).

**TABLE 1:** Summary of the studied species, with information on their body size (ML, maximum mantle length) and reproductive traits (SL, maximum spermatophore length; LL, maximum ligula length; HAL, maximum hectocotylized arm length; AL, maximum arm length; EL, maximum egg length).

**Species**	**ML (mm)**	**SL (mm)**	**LL (mm)**	**HAL (mm)**	**AL (mm)**	**EL (mm)**	**References**
*Abdopus aculeatus*	70	21	1.40	441	490	3	Voight, 2009; Jereb et al., 2014
*Adelieledone piatkowski*	73	38	11.46	182.21	189.8	16	Allcock et al., 2003; Jereb et al., 2014
*Adelieledone polymorpha*	105	41	16.49	207.27	274.6	16	Allcock et al., 2003; Barratt et al., 2008
*Ameloctopus litoralis*	30	6.40	3.60	60.30	270	10	Toll and Voss, 1998; Voight, 2009; Jereb et al., 2014
*Amphioctopus aegina*	100	27	6	240	300	2.4	Huffard and Hochberg, 2005; Voight, 2009; Jereb et al., 2014
*Amphioctopus kagoshimensis*	87	160	6.96	234.90	261	2	Toll and Voss, 1998; Jereb et al., 2014
*Amphioctopus marginatus*	80	80	2.80	192	240	3	Huffard and Hochberg, 2005; Jereb et al., 2014
*Bathypolypus arcticus*	65	64	14.95	84.50	130	18	Muus, 2002; Jereb et al., 2014
*Bathypolypus sponsalis*	70	34	15.40	140	210	15	Muus, 2002; Voight, 2009
*Callistoctopus luteus*	130	87	5.20	468	780	4	Toll and Voss, 1998; Jereb et al., 2014
*Callistoctopus minor*	29	23	6.64	72.50	145	22	Toll and Voss, 1998; Ibáñez et al., 2018
*Callistoctopus ornatus*	130	51	8.97	728	1040	3.5	Toll and Voss, 1998; Voight, 2009; Jereb et al., 2014
*Cistopus chinensis*	100	40	2.40	360	400	15	Jereb et al., 2014
*Cistopus indicus*	90	30	2.70	405	540	4.5	Toll and Voss, 1998; Voight, 2009; Jereb et al., 2014
*Cistopus taiwanicus*	140	33	0.70	525	700	7	Jereb et al., 2014
*Eledone cirrhosa*	150	54	6	360	450	9	Voight, 2009; Jereb et al., 2014
*Enteroctopus dofleini*	600	1130	144	2400	3000	8	Toll and Voss, 1998; Voight, 2009; Jereb et al., 2014
*Enteroctopus megalocyathus*	280	370	61.60	1260	1400	15	Ortiz et al., 2011; Jereb et al., 2014
*Enteroctopus zealandicus*	272	316	53.86	764.32	2067.2	12.5	O’Shea, 1999
*Graneledone antarctica*	45	8	4.50	145	165	–	Voss, 1976; O’Shea, 1999
*Graneledone boreopacifica*	145	131	8.55	1047.48	1218	16	Voss and Pearcy, 1990; Hochberg, 1998; Voight, 2009
*Graneledone challengeri*	145	–	8.55	356.84	433.5	19.5	O’Shea, 1999
*Graneledone taniwha kubodera*	154.5	–	10	44	99	22	O’Shea, 1999
*Graneledone taniwha taniwha*	170	118	12.92	381.99	418.2	24	O’Shea, 1999
*Graneledone verrucosa*	110	100	5.06	325.60	385	17	Allcock et al., 2003; Jereb et al., 2014
*Grimpella thaumastocheir*	50	30	3.50	157.50	225	15	Jereb et al., 2014
*Hapalochlaena fasciata*	50	16	6	112.50	150	9	Voight, 2009; Jereb et al., 2014
*Hapalochlaena lunulata*	50	–	5	80	100	–	Kaneko et al., 2011; Jereb et al., 2014
*Hapalochlaena maculosa*	57	67	7.41	134.52	177.3	9	Toll and Voss, 1998; Jereb et al., 2014
*Megaleledone setebos*	234	100	9.83	702	744.6	41.5	Allcock et al., 2003; Jereb et al., 2014
*Muusoctopus eicomar*	95	79	7.03	281.01	347.7	24	Ibáñez and Cifuentes-Bustamante, 2016; Ibáñez Pardo-Gandarillas, 2016
*Muusoctopus eureka*	110	75	8.80	176.22	587.4	19	Gleadall et al., 2010; Ibáñez Pardo-Gandarillas, 2016
*Muusoctopus januarii*	63	85	5.67	227.99	342.1	19	Allcock et al., 2006; Jereb et al., 2014; Ibáñez Pardo-Gandarillas, 2016
*Muusoctopus johnsonianus*	113	104	9.49	235.04	413.3	–	Allcock et al., 2006; Ibáñez Pardo-Gandarillas, 2016
*Muusoctopus levis*	50	–	4.40	100	142.4	–	Ibáñez Pardo-Gandarillas, 2016
*Muusoctopus longibrachus*	115	70	9.54	296.70	732.5	20	Ibáñez et al., 2006; Ibáñez Pardo-Gandarillas, 2016; Gleadall et al., 2010
*Muusoctopus oregonensis*	93	62	6.23	370.79	420.9	26	Voss and Pearcy, 1990; Ibáñez Pardo-Gandarillas, 2016
*Muusoctopus profundorum*	67	–	2.33	201	268	–	Gleadall et al., 2010; Ibáñez Pardo-Gandarillas, 2016
*Muusoctopus rigbyae*	105	104	16.80	315	420	24	Vecchione et al., 2009
*Muusoctopus tangaroa*	100	120	18.50	202	494	23	O’Shea, 1999
*Muusoctopus thielei*	65	–	8.45	143	175.5	–	Ibáñez Pardo-Gandarillas, 2016
*Muusoctopus yaquinae*	83	136.1	9.79	198.54	258.2	12	Voss and Pearcy, 1990; Ibáñez Pardo-Gandarillas, 2016
*Octopus bimaculatus*	200	35	1.40	700	1000	4	Jereb et al., 2014
*Octopus bimaculoides*	120	33	2.76	336	420	18	Jereb et al., 2014
*Octopus californicus*	140	70	30.80	396.90	490	17	Hochberg, 1998; Jereb et al., 2014
*Octopus campbelli*	36	–	6.60	78	104	1.7	O’Shea, 1999
*Octopus conispadiceus*	166	80	33.20	398.40	498	28	Toll and Voss, 1998; Jereb et al., 2014
*Octopus cyanea*	172	48	3.44	928.80	1032	3	Toll and Voss, 1998; Voight, 2009; Jereb et al., 2014
*Octopus fitchi*	29	19	–	–	–	–	Jereb et al., 2014
*Octopus hongkongensis*	200	200	28	644	164	–	Toll and Voss, 1998; Ibáñez et al., 2018
*Octopus huttoni*	57	39	9.80	129	180	3.1	O’Shea, 1999
*Octopus insularis*	120	35	2.04	453.60	504	1.5	Leite et al., 2008; Jereb et al., 2014
*Octopus kaurna*	85	88	6.80	416.50	595	11	Toll and Voss, 1998; Jereb et al., 2014
*Octopus laqueus*	24	22	0.62	57.36	106.8	2.6	Kaneko and Kubodera, 2005; Voight, 2009
*Octopus maya*	250	56	4.75	870	1125	17	Voss and Toll, 1998; Jereb et al., 2014
*Octopus mernoo*	85	–	15.47	105.40	168.1	23.5	O’Shea, 1999
*Octopus mimus*	155	58	2.79	716.10	930	3.2	Voight, 2009; Jereb et al., 2014
*Octopus oliveri*	69	34	1.52	224.25	273.1	7.5	Toll and Voss, 1998; O’Shea, 1999
*Octopus pallidus*	150	173	24	355.05	394.5	13	Toll and Voss, 1998; Jereb et al., 2014
*Octopus parvus*	40	12	2	120	280	1.8	Toll and Voss, 1998; Ibáñez et al., 2018
*Octopus rubescens*	100	60	11	405	450	4	Hochberg, 1998; Jereb et al., 2014
*Octopus salutii*	125	74	20.63	953.75	1148.7	6	Mangold, 1998; Toll and Voss, 1998; Voight, 2009
*Octopus tehuelchus*	60	57	2.70	180	240	15	Ré, 1998; Toll and Voss, 1998; Alves and Haimovici, 2011; Jereb et al., 2014
*Octopus tetricus*	135.5	24	4.10	418	547	3	O’Shea, 1999; Ibáñez et al., 2018
*Octopus vulgaris*	250	65	5.25	180.50	1375	2.7	Mangold, 1998; Toll and Voss, 1998; Voight, 2009; Jereb et al., 2014
*Octopus wolfi*	15	8.7	1.50	–	99	–	Toll and Voss, 1998
*Pareledone aequipapillae*	63	94	5.54	90.97	133.2	20	Allcock, 2005
*Pareledone albimaculata*	38	46	3.80	59.01	81.4	10	Allcock, 2005
*Pareledone aurata*	49	55	5.54	61.98	81.3	11	Allcock, 2005
*Pareledone charcoti*	70	61	7.98	66.99	128.1	13	Kubodera and Okutani, 1994; Allcock, 2005
*Pareledone cornuta*	60	71	5.58	64.02	108.8	20	Allcock, 2005
*Pareledone felix*	42	70	4.75	66.99	69.5	22	Allcock et al., 2007; Voight, 2009
*Pareledone panchroma*	41	37	4.26	50.02	56.8	14	Allcock, 2005
*Pareledone serperastrata*	36	52	3.06	59.00	62.1	7	Allcock, 2005
*Pareledone subtilis*	44	42	4.4	49.98	67.1	14	Allcock, 2005
*Pareledone turqueti*	60	72	8.5	–	250	19.8	Daly and Rodhouse, 1994; Allcock et al., 2007; Barratt et al., 2008
*Paroctopus digueti*	42	22	3.36	107.10	126	10	Jereb et al., 2014
*Pinnoctopus cordiformis*	310	228	15.50	686.34	261	7	Voight, 2009; Jereb et al., 2014
*Praealtus paralbida*	65	120	3.05	220.02	221	–	Allcock et al., 2004; Jereb et al., 2014
*Robsonella fontaniana*	69	50	6.90	258.75	345	5	Toll and Voss, 1998; Ibáñez et al., 2008; Jereb et al., 2014
*Scaeurgus unicirrhus*	90	84	9.90	56.34	405	3	Mangold, 1998; Toll and Voss, 1998; Voight, 2009; Jereb et al., 2014
*Thaumeledone gunteri*	50	50	8.45	85	100	10	Allcock et al., 2004; Jereb et al., 2014
*Thaumeledone peninsulae*	48	45	4.70	51.02	70.1	13	Allcock et al., 2004
*Thaumeledone rotunda*	62	74.40	14	87	127	16	Allcock et al., 2004
*Thaumeledone zeiss*	55	–	9.35	58.08	79.5	9.3	O’Shea, 1999
*Velodona togata*	180	174	16.20	688.50	810	19	Jereb et al., 2014
*Vulcanoctopus hydrothermalis*	60	54	5.70	168	240	5.5	González et al., 1998, 2002

### Statistical Analyses

We performed three complementary analyses to determine how the variation in reproductive traits is related to phylogenetic history. First, we performed univariate analyses to estimate the amount of phylogenetic signal (λ) and the mode of evolution (κ) of each trait (ML, HAL, LL, and SL) employing BayesTraits v3 (Meade and Pagel, 2017). Second, we ran three separate variable-rates regression models (Baker et al., 2016) to determine how HAL, LL, and SL vary as a function of body size (ML), and also diagnose in which lineages these structures have diverged more than expected after controlling for size effects. Third, we performed a phylogenetic principal component analysis (PCA) to determine the degree of covariation between reproductive traits and study correlated evolution between them after removing body size effects.

Phylogenetic signal in our univariate analyses was estimated employing Pagel’s λ, which quantifies the tendency of closely related lineages to resemble each other in comparison to a Brownian motion model of evolution (Pagel, 1999, 2002): λ = 1 indicates that the distribution of the phenotypic traits along the tips of the phylogeny closely resemble the expectation based on Brownian motion (i.e., high phylogenetic signal), whereas λ = 0 shows that patterns of phenotypic resemblance due to shared phylogenetic history is negligible (i.e., low phylogenetic signal). The mode of phenotypic evolution was estimated using Pagel’s κ, which scales the branch lengths between their original values and a single constant, mimicking gradual evolution when κ = 1, and a punctuated model of evolution when κ = 0 (Pagel, 1999, 2002). The posterior distribution of all parameters was visualized in the software Tracer V1.6 (Rambaut et al., 2013). To test if λ and κ estimated values were different from pre-established values, we first estimated the higher posterior distribution of these parameters for each trait in BayesTraits V3, forcing each parameter to have a value of 0 for λ (i.e., no signal) and 0 and 1 for κ (i.e., perfectly punctuated or perfectly gradual evolution, respectively) and compared the fit of estimated vs. forced models with log_10_ Bayes Factor (BF). The larger the BF value, the better the fit of the estimated model in comparison against the forced one, with BF > 0.5 being generally interpreted as strong support for the estimated model and BF > 1 being considered decisive (Kass and Raftery, 1995). Because these univariate analyses include scaling effects, we expect HAL, LL, and SL to exhibit less signal (i.e., a lower λ) than ML if they evolve faster than this trait due to an evolutionary arms race (i.e., signal is expected to decrease if traits diverge fast in response to selection).

To estimate phenotypic selection on reproductive traits after removing potential scaling effects, we performed three separate variable-rates regressions with log_10_-transformed HAL, LL, and SL as a function of ML. This regression model was recently developed by Baker et al. (2016) and allows the rate of change to vary through the phylogenetic branches and identifies areas of the tree where the rate of evolution departs significantly from background levels (Venditti et al., 2011). With this purpose, this regression method estimates a branch-specific metric Δ_V_/Δ_B_ that contrasts the expected phenotypic variance Δ_V_ along the branch due to changes in evolutionary rates (i.e., acceleration or deceleration) vs. the expectation attributable to the background evolutionary rate (Δ_B_). Branches in which the amount of estimated phenotypic change doubles the background rate (Δ_V_/Δ_B_ > 2) constitute regions of the phylogeny that were likely under positive selection (Baker et al., 2016). We implemented the variable-rates regression model with the phylogenetic independent contrast regression module in BayesTraits, employing the reversible-jump Markov chain Monte Carlo (RJMCMC) to determine whether Δ_V_/Δ_B_ > 2 results were observed in more than 95% of the posterior distribution. In all Bayesian analyses described above, we ran 20,000,000 iterations via the MCMC method. Parameters were sampled every 1,000 iterations, excluding the first 25% of iterations. The 95% highest posterior density (95% HPD) for each parameter was calculated in Tracer, and all analyses performed in BayesTraits; outgroup and sister groups were excluded.

To determine to what extent the different reproductive traits studied here evolve in tandem, we performed a phylogenetic PCA (Revell, 2009) including log_10_-transformed ML, HAL, LL, and SL. To account for phylogenetic signal, λ was estimated concomitantly with parameters from the PCA. Because we were primarily interested in identifying potentially contrasting evolutionary strategies between lineages, we focused on the second and third principal components (PC2 and PC3) that provide information on differences in morphology or shape after removing size effects embedded in the first principal component.

Finally, to explore the association response to selection on other traits, the correlation between reproductive traits [spermatophore length (SL) and egg length (EL)] and morphological traits [arm length (AL) and HAL] were analyzed using phylogenetic generalized least squares (PGLS) regressions (Pagel, 1999). To account for phylogenetic signal, λ was estimated concomitantly with parameters from the regression model (Pagel, 2002). After reviewing different sources, egg length data was obtained only for 72 species (Table 1).

## Results

Among the species in our dataset, ML exhibited a 40-fold variation, with values ranging between 15 mm in *Octopus wolfi* to 600 mm in *Enteroctopus dofleini*. By contrast, reproductive traits tended to exhibit a higher variation between extremes, from 54-fold variation in HAL (i.e., 44–2,400 mm), 176-fold in SL (i.e., 6.4– 1,130 mm), and 257-fold in LL (0.56 – 144 mm) (see Table 1).

All traits exhibited a high phylogenetic signal, with λ > 0.75 statistically different from λ = 0, as indicated by BF > 11 in all traits (Table 2). As hypothesized (see section “Materials and Methods”), λ estimated for reproductive traits were generally lower than values for ML, agreeing with the observation that these traits exhibited more variation across species than ML. Regarding the mode of evolution inferred by κ, calculated in combination with λ in the univariate analyses, estimates for ML, HAL, LL, and SL were intermediate between κ = 0 and 1 and statistically different from those values according to BF estimates (BF > 1.39 for all comparisons) (Table 2). This implies that all traits evaluated tended to evolve slower than predicted (i.e., evolutionary stasis) in longer branches when compared to shorter branches (Pagel, 1999, 2002).

**TABLE 2:** Phylogenetic signal (λ) and evolutionary mode (κ) obtained in univariate analyses.

	**Lambda (λ)**	**BF (λ > 0)**	**Kappa (κ)**	**BF (κ > 0)**	**BF (κ < 1)**
ML	0.91(0.77-0.98)	12.05	0.55(0.29-0.82)	5.76	4.25
HAL	0.86(0.67-0.97)	11.58	0.52(0.24-0.83)	3.23	3.20
LL	0.87(0.75-0.96)	28.23	0.22(0.01-0.45)	11.92	13.47
SL	0.75(0.45-0.96)	11.15	0.61(0.31-0.93)	1.39	1.41

*Numbers in parentheses correspond to the highest posterior density (95% HDP) and represent the 95% credibility confidence estimated with a Bayesian approach, whereas the Bayes factor (BF) provides the weight of the evidence.*

**FIGURE 3:**
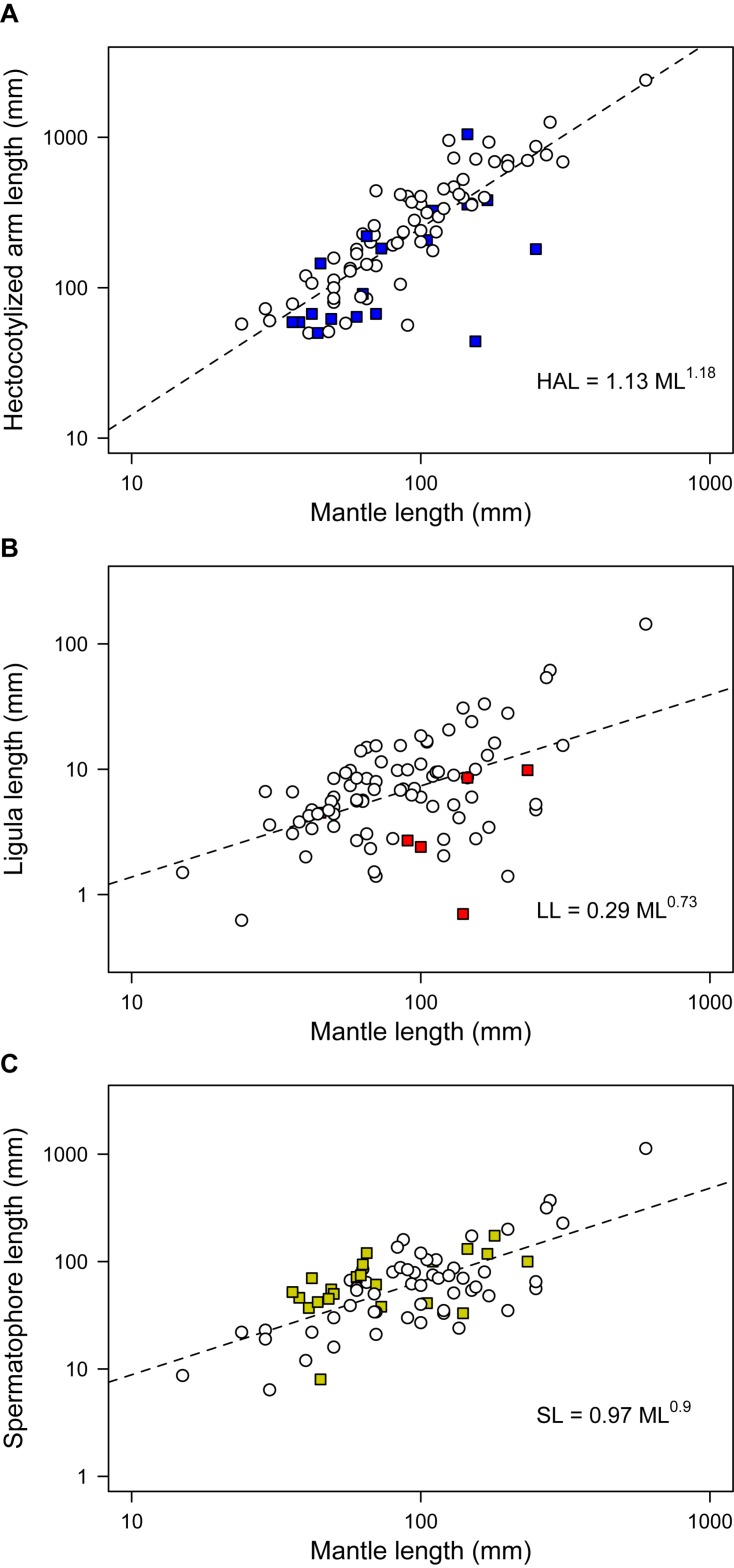
Scaling of morpho-functional reproductive traits in benthic octopuses. Mantle length is plotted against **(A)** hectocotylized arm length, **(B)** ligula length, and **(C)** spermatophore length. Colored symbols correspond to the lineages where we detected positive selection – i.e., more divergence than expected based on background rates of phenotypic evolution – according to a variable-rates regression model. Colors as in Figure 2.

Phylogenetic regressions of reproductive traits as a function of ML indicated that all variables scaled positively with body size, with scaling exponents corresponding to 1.18 for HAL (95% HDP between 0.99 – 1.36), 0.73 (0.53 – 0.95) for LL, and 0.90 (0.71 – 1.10) for SL (Figure 3). Consequently, HAL tends to become disproportionally larger as size increases, whereas SL scales roughly isometrically, and LL is relatively shorter in larger lineages. According to variable-rates phylogenetic regressions controlling for these scaling effects, several regions of the phylogeny exhibited accelerated rates of phenotypic evolution, and met the criterion of Δ_V_/Δ_B_ > 2 proposed as evidence of positive selection (Figure 2). This was particularly true for HAL and SL, for which we detected selection in a total of 33 and 44 branches, respectively, or roughly 20 to 30% of all branches (Table 3). Interestingly, separate analyses for both traits gave rise to similar qualitative results, suggesting accelerated rates of phenotypic divergence for these traits in Antarctic, and deep-sea octopuses from the family Megaleledonidae (Figures 2B,D). In contrast, evidence of positive selection in LL was limited to only 8 branches, or 4.9% of the total (Table 3), most of them involving the *Cistopus* clade (Figure 2C).

**TABLE 3:** Results of the variable-rates model for positive selection over three reproductive traits of benthic octopuses.

	**Total**	**Mean Δ_V_/Δ_B_**	**Branches under**	**Mean Δ_V_/Δ_B_ > 2**
	**branches**	**(±SD)**	**selection**	**(±SD)**
HAL ∼ ML	160	3.04 ± 5.33	33	10.39 ± 8.43
LL ∼ ML	164	1.47 ± 2.16	8	9.20 ± 6.04
SL ∼ ML	150	2.03 ± 1.21	44	3.84 ± 0.52

**FIGURE 4:**
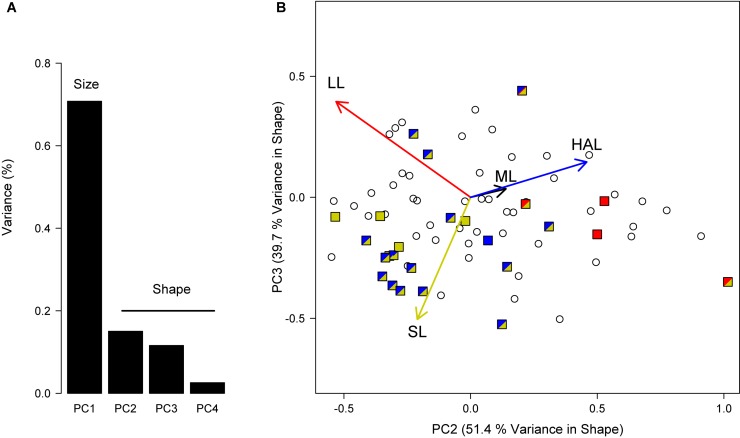
Phylogenetic principal component analysis (PCA) to study the correlated evolution between reproductive traits. **(A)** We included log_10_-transformed ML, HAL, LL and SL, removed PC1 that encompassed primarily scaling effects, and worked with the remaining components that indicate differences in “shape” across lineages. **(B)** Species under positive selection according to variable-rates regression are shown in colors, as in Figures 2, 3. Note that selection was detected in more than a single trait in several lineages.

The phylogenetic PCA including log_10_-transformed ML, HAL, LL, and SL strongly supported correlated evolution between these reproductive traits (Figure 4). As expected, PC1 accounted for a substantial fraction of the variance in the original data (70.8%), which could be attributed to a variation associated with body size, whereas the remaining PCs involve phenotypic variation that is independent of size (i.e., “shape” for simplicity; Figure 4). Accordingly, ML loadings in the remaining PCs were very low because most variation in this trait was explained by PC1. After removing the effects of size, PC2, and PC3 combined accounted for 91.1% of the variance in shape observed across lineages, with loadings indicating that most of the variance in HAL is explained by PC2 and in SL by PC3, with LL falling somewhere in between (Figure 4). Contrasting these results against the outcome of the variable-rates regressions, we can identify two distinct groups (Figure 4), one exhibiting reduced hectocotyli and large spermatophores (low HAL and high SL), and the other with relatively large hectocotyli with small ligulae (high HAL and low LL). As clearly illustrated in Figure 4, results from variable-rates regressions performed separately for each reproductive trait provided very consistent results and complementary evidence of positive selection across the same phylogenetic lineages.

Spermatophore length did not correlate with egg length (*n* = 72 species, *r* = 0.050, 95% HPD between 0.0013 and 0.1082, λ = 0.85, Supplementary Figure S1). Interestingly, the correlation between arm length and hectocotilized arm length was higher than zero (*n* = 85 species, *r* = 0.8124, 95% HPD between 0.7946 and 0.8372, λ = 0.75, Supplementary Figure S2).

## Discussion

The present results support our original hypothesis on reproductive traits in male benthic octopuses, evidencing that spermatophores, and hectocotyli (arm and ligula) exhibited accelerated rates of evolution, at least in several Antarctic and deep-water lineages (Megaleledonidae and *Cistopus*), presumably due to sexual selection. All reproductive traits showed a fold-range of morphological variation that is substantially larger than expected based solely on differences in body size (i.e., ML), and variable-rates regression analyses clearly indicated that several lineages tended to deviate substantially from allometric expectations. While our analyses focussed primarily on the variation in reproductive traits after statistically removing the effects of size (effectively using levels of ML divergence between lineages as a standard of comparison), body size may have also been under selection in some lineages within Octopodoidea, as observed in other clades exhibiting sexual dimorphism in size (e.g., Amphitretidae and Tremoctopodidae Jereb et al., 2014). Unfortunately, this possibility cannot be directly tested with our phylogenetic comparative approach in the absence of precise information on most aspects of reproductive behavior of the analyzed species, including competition for mates, mate choice and mating position, as well as their intraspecific body size variation. Additionally, other environmental variables and selective pressures may have contributed to body size evolution of many of these lineages. Nonetheless, it is important to consider that body size may also evolve in response to sexual selection and gametic competition. For instance, we detected a clear positive association between ML and SL, indicating that larger species – and presumably larger individuals within a species – have bigger spermatophores and consequently more sperm to transfer to females. Accordingly, this same trend was previously described by Voight (2009); therefore, we do not only provide support for such finding within a strict phylogenetic context, but we also detected which groups and lineages deviated from allometric expectations (see below).

Admittedly, while our analyses provided strong evidence of selection in several regions of the phylogeny, some limitations must be highlighted. First, note that our phylogenetic analysis detects regions of the phylogeny with extraordinary rates of evolution in comparison to background rates inferred from the same dataset, which is inherently conservative and can only detect selection in restricted regions of the phylogeny. Therefore, it is possible that we might have missed other evolutionary clades whose phenotypic diversification might be partly explained by selection (while decreasing the Δ_V_/Δ_B_ > 2 might partly circumvent this problem, it would also increase the type I error). Second, our analyses do not inform specifically on the mechanisms that underlie these results. Consequently, alternative adaptive scenarios must be taken into account to determine the likelihood that observed patterns emerge from sexual selection. In this context, we believe that two possibilities are worth considering: (1) results reflected adaptation to environmental conditions, and/or (2) they partly reflected selection on correlated traits. With regard to the first scenario, the high evolutionary rates (i.e., Δ_V_/Δ_B_ > 2) in HAL and SL involved primarily lineages of Antarctic and deep-sea octopuses from the family Megaleledonidae. Because ectotherm organisms inhabiting cold waters tend to exhibit lower fecundity, slower growth rates, and larger life-spans (van Voorhies, 1996; Ibáñez et al., 2018), it is plausible to expect that cold-adapted lineages may evolve larger spermatophores compared to warm-water species (Voight, 2009). Moreover, among deep-sea organisms that live in low densities the probability of a mating encounter is reduced (see Hoving et al., 2012), and therefore, selection may favor a high reproductive investment per mating. Consequently, we suggest it is possible that the colonization of cold-waters and deep-sea habitats might partly explain the high evolutionary rates detected in this clade (Megaleledonidae) and their larger spermatophores. Alternatively, it is also possible that some of the patterns detected reflect correlated responses to selection on other traits, which might be particularly true for HAL given its close association with AL (*r* = 0.81). All lineages detected in the variable-rates regression for HAL exhibited smaller arms than predicted from allometry, though it is not clear exactly which selective pressures might favor smaller arms.

Importantly, while these alternative evolutionary scenarios might justify why members of the family Megaleledonidae differed from other groups, they failed to explain the extremely high diversity within this clade and its degree of phenotypic variation (i.e., HAL). Within Antarctic octopods, the family Megaleledonidae is the most diverse, with new species still being discovered (Xavier et al., 2018), and here we show that this highly speciose group also exhibited extremely elevated levels of phenotypic divergence in male reproductive traits (i.e., SL). We contend that the speciation rates observed in this clade in conjunction with the extremely high rates of phenotypic evolution cannot be explained by niche diversification, and likely reflect sexual selection (i.e., “runaway” sexual selection), where the coevolution of female mating preferences and male sexual characters promotes reproductive isolation and foments speciation (see Lande, 1982). The process of sperm competition has been well described in benthic octopuses, highlighting the role of cephalopod behavior in mediating intra-sexual competition (e.g., several males attempting to mate with a female simultaneously; reviewed by Hanlon and Messenger, 1996). Similarly, the occurrence of multipaternity has also been described in some species of octopuses, suggesting that females are able to fertilize eggs with the sperm of multiple males, decreasing the probability of fertilizing high number of eggs with the sperm of single male, as reported for *Graneledone boreopacifica* (Voight and Feldheim, 2009), *Octopus vulgaris* (Quinteiro et al., 2011), *E. dofleini* (Larson et al., 2015), *O. minor* (Bo et al., 2016), *Hapalochlaena maculosa* (Morse et al., 2018), and *O. oliveri* (Ylitalo et al., 2019). Additionally, it has been proposed (but not verified by other authors, nor by our own data) that species with large-sized ligula do not only use this structure for spermatophores transfer, but also to breakdown or modify the position of spermatophores from rival males (Cigliano, 1995; Hanlon and Messenger, 1996; Norman et al., 2004). Nonetheless, this behavior has been described in other taxa (such as insects) that use their copulatory organs to extract the sperm left by other males as a mechanism to counteract sperm competition (Birkhead and Moller, 1999). Finally, cryptic female choice favoring larger spermatophores has been reported in the sepiolid squid *Idiosepious paradoxus* through postcopulatory behavior (see Sato et al., 2013), and may therefore be taking place in closely related octopods.

While these studies leave no doubt that sexual selection is potentially an important factor shaping the evolution of benthic octopuses, the fact that this seems to be particularly the case for members of the family Megaleledonidae remains an open question. It is possible that this group has evolved mating strategies and reproductive habits that exacerbate sperm competition via, for instance, territoriality or female postcopulatory selection. Interestingly, Rocha et al. (2001) speculated that the wide range of sizes of maturing ova described in the megaleledonids *Pareledone charcoti* and *Adelieledone polymorpha* could indicate repeated spawning in these species, contrasting with the majority of octopods that are considered terminal spawners. Another potential explanation that is not mutually exclusive corresponds to that, due to the environmental conditions encountered by these Antarctic deep-sea species (i.e., temperature and environmental stability; see Ibáñez et al., 2018), the impact of sexual selection become disproportionally important in this group in comparison to other benthic octopuses. Indeed, the lack of correlation between SL and EL suggest the absence of environmental selection on SL at the poles or at deep water environments.

In other words, we speculate that other factors shaping phenotypic evolution, such as predation, interspecific competition or environmental heterogeneity, may be relatively less important in the Antarctic deep-sea species. Perhaps the combined action of these two phenomena, namely the evolution of exclusive reproductive strategies in this clade in response to specific environmental pressures, may ultimately explain the very strong signal of selection and phenotypic divergence detected across males of this family. Overall, our phylogenetic approach provides some evidence of sexual selection within benthic octopuses, particularly for Megaleledonidae, and a potentially relevant role in their diversification. Detailed studies on different mating behaviors and how they relate with morphological and life-history traits are still necessary to better understand the adaptation of different cephalopod lineages to highly contrasting environments worldwide.

## Data Availability Statement

Publicly available datasets were analyzed in this study. This data can be found here: https://data.mendeley.com/datasets/5vkm46hm49/1.

## Author Contributions

CI, ER, and MP-G conceived the idea, designed the study, analyzed the data, and led the writing of the manuscript. SC, JP-Á, and JC collaborated in literature review, writing, and provided the editorial advice. All authors have read and commented on the manuscript.

## Conflict of Interest

The authors declare that the research was conducted in the absence of any commercial or financial relationships that could be construed as a potential conflict of interest.
