# Supplementary material for: Sexual Selection and the Evolution of Male Reproductive Traits in Benthic Octopuses
Source: Front Physiol. 2019 Oct 9;10:1238. doi: 10.3389/fphys.2019.01238 (PMC6794433; doi:10.3389/fphys.2019.01238)
Supplement: Supplementary file 1 [file Table_1.DOCX]

Supplementary Material


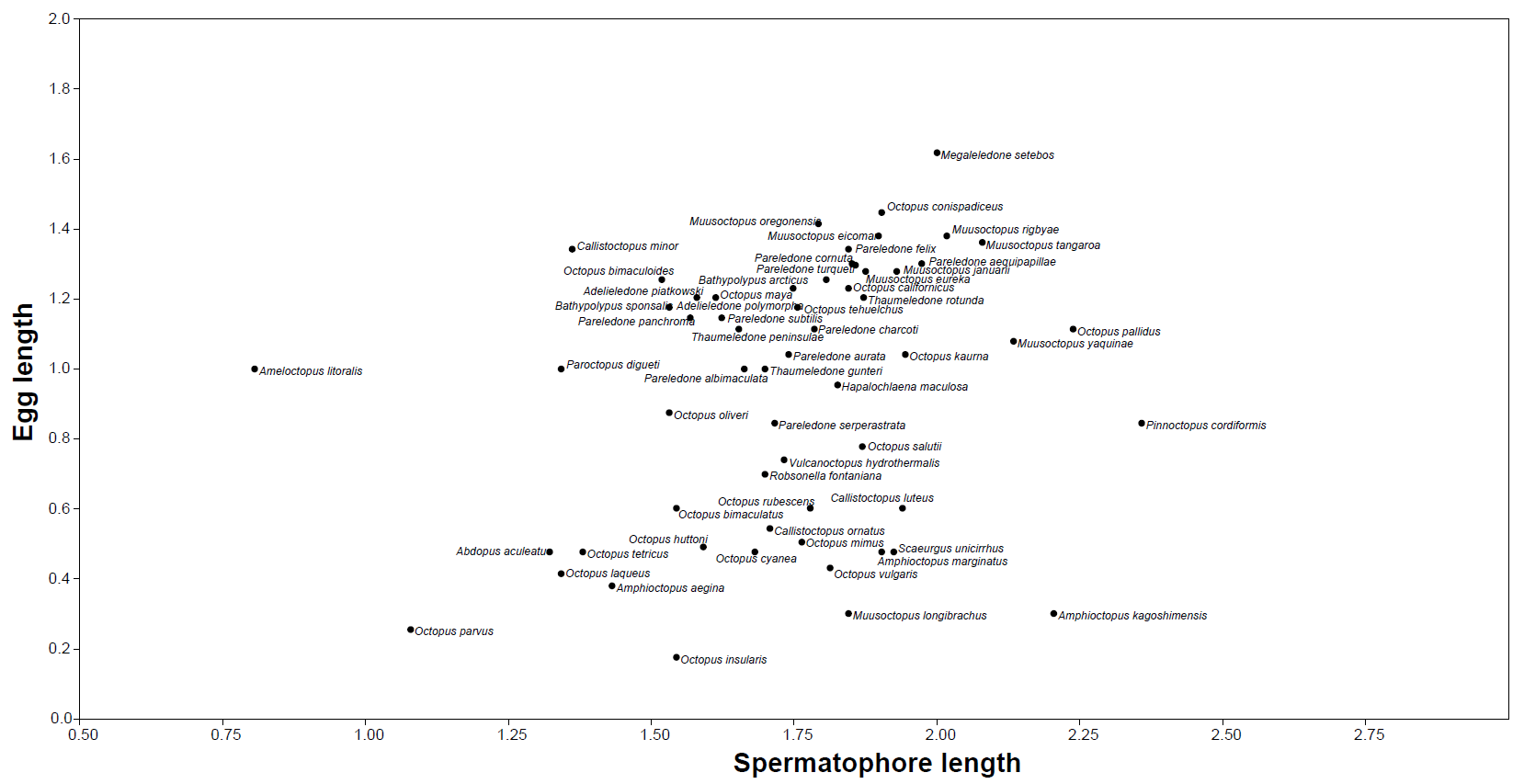


**Supplementary Figure 1.** Relationship between (log) spermatophore length and (log) egg length of benthic octopuses.


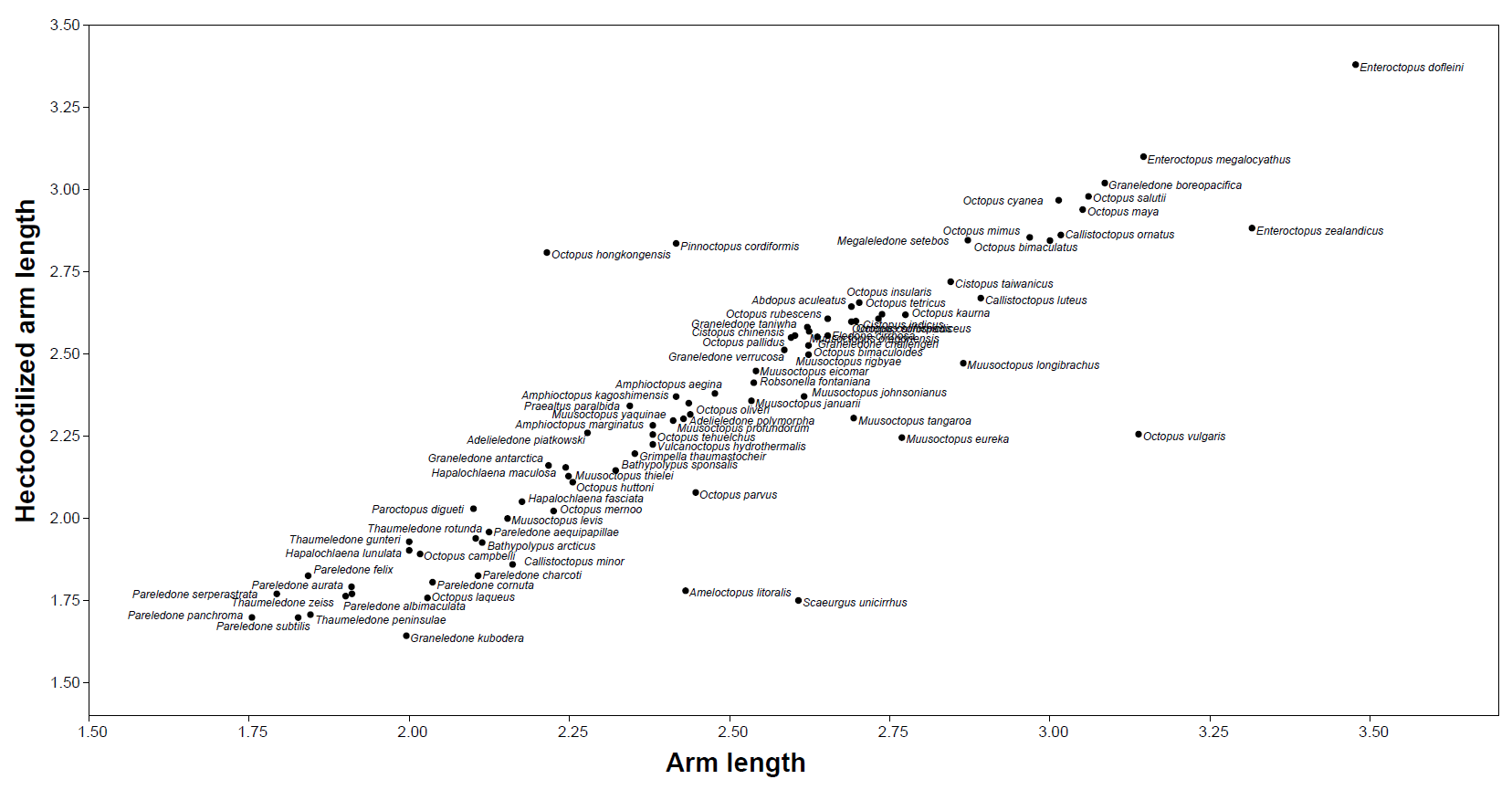


**Supplementary Figure 2.** Relationship between (log) arm length and (log) hectocotilized arm length of benthic octopuses.
